# The Combination of RAD001 and MK-2206 Exerts Synergistic Cytotoxic Effects against PTEN Mutant Gastric Cancer Cells: Involvement of MAPK-Dependent Autophagic, but Not Apoptotic Cell Death Pathway

**DOI:** 10.1371/journal.pone.0085116

**Published:** 2014-01-09

**Authors:** Dongmei Ji, Zhe Zhang, Lei Cheng, Jinjia Chang, Shanshan Wang, Biqiang Zheng, Rongliang Zheng, Zuojun Sun, Chenchen Wang, Zhiqing Zhang, Rujiao Liu, Xiaowei Zhang, Xin Liu, Xiaofeng Wang, Jin Li

**Affiliations:** 1 Department of Medical Oncology, Fudan University Shanghai Cancer Center, Shanghai Medical College, Fudan University, Shanghai, China; 2 Department of Interventional Radiology, Suzhou Hospital Affiliated to Nanjing Medical University, Suzhou, Jiangsu, China; 3 Department of Gastric Cancer and Soft Tissue Sarcomas, Fudan University Shanghai Cancer Center, Shanghai Medical College, Fudan University, Shanghai, China; 4 Institute of Neuroscience, Soochow University, Suzhou, Jiangsu, China; Complutense University, Spain

## Abstract

In the current study, we showed that the combination of mammalian target of rapamycin (mTOR) inhibitor RAD001 (everolimus) and Akt inhibitor MK-2206 exerted synergistic cytotoxic effects against low-phosphatase and tensin homolog (PTEN) gastric cancer cells (HGC-27 and SNU-601 lines). In HGC-27 cells, RAD001 and MK-2206 synergistically induced G1/S cell cycle arrest, growth inhibition, cell death but not apoptosis. RAD001 and MK-2206 synergistically induced light chain 3B (LC3B) and beclin-1 expression, two important autophagy indicators. Meanwhile, the autophagy inhibitor 3-methyladenine (3-MA) and chloroquine inhibited the cytotoxic effects by RAD001 and MK-2206, suggesting that autophagic, but not apoptotic cell death was important for the cytotoxic effects by the co-administration. We observed that the combination of RAD001 and MK-2206 exerted enhanced effects on Akt/mTOR inhibition, cyclin D1 down-regulation and ERK/MAPK(extracellular signal-regulated kinase/mitogen-activated protein kinases) activation. Intriguingly, MEK/ERK inhibitors PD98059 and U0126 suppressed RAD001 plus MK-2206-induced beclin-1 expression, autophagy induction and cytotoxicity in HGC-27 cells. In conclusion, these results suggested that the synergistic anti-gastric cancer cells ability by RAD001 and MK-2206 involves ERK-dependent autophagic cell death pathway.

## Introduction

Extensive translational research and development of experimental therapeutics on gastric cancers have been achieved recently, however, there has been no significant improvement in overall survival for gastric cancer patients [1], [2], [3]. One key hurdle is the molecular heterogeneity of gastric cancers, which impedes uniform application of specific molecularly targeted agents [1], [2], [3]. One important pathway that is frequently dysregulated is phosphatidylinositol 3-kinase (PI3K)/protein kinase B (PKB or Akt)/mammalian target of rapamycin (mTOR) signaling cascade [4], [5], which is activated by various growth factor receptors (i.e. HER2(human epidermal growth factor receptor-2) [6]) or by phosphatase and tensin homolog (PTEN) mutation [7].

The activation of the PI3K/Akt signaling pathway is frequently associated with tumorigenesis and cancer progression [4], [8]–[10]. Furthermore, dysregulated PI3K/Akt signaling may contribute to tumor resistance to a variety of anti-neoplastic agents [11]. Various inhibitors designed to specifically target this pathway are now being developed for clinical use [4], [8], [12], and the combination of these inhibitors with chemotherapy has successfully attenuated chemotherapeutic resistance in gastric cancer cell lines [4].

RAD001 (everolimus) is a derivative of rapamycin and is functionally similar to rapamycin as an allosteric inhibitor of mTOR. RAD001 improves progression-free survival of patients with renal cell cancer and has therefore been approved by the US Food and Drug Administration (FDA) for this indication [13]. In many other solid malignancies, however, RAD001 exerts modest anti-cancer effects that though promising, are not sufficient to warrant monotherapy [14], [15]. RAD001, just like rapamycin, causes Akt activation in human cancer cells while inhibiting the mTOR signaling [16], [17], serving as one key resistance factor [17]. Recent studies have been focusing on the enhanced anti-cancer efficacy of the combination of RAD001 with an Akt inhibitor [17].

MK-2206, an orally bio-available allosteric inhibitor of Akt, has shown potential anti-neoplastic activity [11], [18]. MK-2206 directly inhibits the activity of Akt in a non-ATP competitive manner, which results in the inhibition of the Akt signaling pathway and tumor cell proliferation [11], [18]. In the current study, we investigated the potential effects of MK-2206 to overcome RAD001 resistance in PTEN mutant gastric cancer cells. Our results showed that the combination of RAD001 and MK-2206 exerted synergistic anti-cancer activity against PTEN mutant gastric cancer cell lines, and suggested that ERK mitogen-activated protein kinases (MAPK)-dependent autophagy pathway, but not apoptosis mediated this process.

## Materials and Methods

### Chemical and reagents

RPMI-1640, fetal bovine serum (FBS) and serum-free opti-MEM were obtained from Gibco (Carlsbad, CA). Lipofectamine™ 2000 transfection reagent and PLUS™ reagent were purchased from Invitrogen (Shanghai, China). Goat anti-rabbit and mouse horseradish peroxidase (HRP)-conjugated IgG were purchased from Santa Cruz biotechnology (Santa Cruz, CA). All other phospho- and non-phospho- antibodies were purchased form Cell Signaling Tech (Denver, MA). The enhanced chemiluminescence (ECL) western blot reagent kit was purchased from Pierce (Rockford, IL). 3-methyladenine (3-MA) and chloroquine were purchased from Sigma (St. Louis, MO). PD98059, U0126 and z-VAD-fmk were purchased from Calbiochem (Shanghai, China), C6-ceramide was obtained from Avanti (Alabaster, AB).RAD001 was kindly provided by Novatis, MK2206 was ordered from selleckchem.com (Shanghai, China).

### Cell culture

Gastric cancer cell lines HGC27, AGS, SNU-601 and NCI-N87 were purchased from Cell Resource Center of Shanghai Institute of Life Sciences, Chinese Academy of Sciences [19], [20]. Other cell lines including S7901,MKN28,MKN45,GES-1 were established as described in our previous publications [19], [20]. Gastric cancer cell lines AGS, MNK-45, HGC-27, MKN-28, SGC-7901, N-87 and human gastric mucosal epithelial cell line GES-1 were maintained in RPMI 1640 or F12K (Gibco Life Technologies, Carlsbad, CA) or MEM supplemented with 10% FBS, penicillin/streptomycin (1∶100, Sigma), and 4 mM L-glutamine and 0.19% HEPES (Sigma), in a humidified incubator at 37°C and 5% CO_2_.

### “Dead” cell detection by trypan blue staining

The number of dead HGC-27 cells (trypan blue positive) after indicated treatment was recorded, and the percentage (%) of dead cells was calculated by the number of the trypan blue stained cells divided by the total number of cells.

### Protein isolation and western blot

After indicated treatments, the cells were washed with ice-cold PBS and then lysed using lysis buffer (pH 7.4) containing 1% Nonidet P-40, 1% deoxycholate, 0.1% sodium dodecyl sulfate, 150 mmol/L sodium chloride and 10 mmol/L Tris-HCl. The lysates were collected and centrifuged. The concentration of the extracted protein was measured by bicinchoninic acid assay kit (Sigma-Aldrich, catalogue B9643, USA). The extracted protein was boiled for 5 min in 5× loading buffer. Samples were separated on 10% SDS-polycrylamide gel, and after electroblotting onto polyvinylidene fluoride(PVDF) membranes (Millipore, USA), the membranes were blocked with blocking solution [10% (w/v) milk in Tris-bufferedsolution plus Tween-20 (TBST): 50 mM Tris–HCl, 150 mM NaCl, pH = 7.5, 0.1% (v/v) Tween 20], incubated overnight at 4°C with primary antibodies, and then incubated with HRP-conjugated anti-rabbit/mouse IgG. Detection was performed by ECL using Supersingnal West Pico Chemiluminescent Substrate according to the manufacturer's instructions. Band intensity was quantified by densitometry using ImageJ software and was normalized to non-phosphorylation kinases or loading controls. Quantification value was expressed as the fold change vs. band numbered “1.00”. ImageJ was downloaded from NIH website (http://rsbweb.nih.gov/ij/download.html).

### Flow cytometric analyses of cell cycle distribution

After indicated treatments, both detached and adherent HGC-27 cells were collected and centrifuged at 1000 g for 5 min at 4°C. Pellets were rinsed with ice-cold PBS and fixed with 70% ethanol for 2 hours. Cells were then stained with staining buffer (PBS containing 20 µg/ml of propidium iodide (PI), 100 µg/ml RNase A, and 0.1% Triton X-100) for 15 min at 37°C in the dark. Cell cycle distribution in these cells was then analyzed by a flow cytometer (BD Bioscience).

### CCK-8 cell viability assay

Cell viability after indicated treatment/s was measured by Cell Counting Kit-8 (CCK-8) (Dojindo,Japan) assay according to manufacturer's protocol as reported [21]. The OD value of group received the indicated treatment was normalized to OD value of untreated control group.

### Analysis cell apoptosis by fluorescence-activated cell sorting (FACS) sorting PE-Annexin V positive cells

After the indicated treatment, HGC-27 cell apoptosis was detected by the PE Annexin V Apoptosis Detection Kit (BD Bioscience, Shanghai, China) according to the manufacturer's protocols. Briefly, HGC-27 cells were stained with PE-Annexin V and 7-AAD (Molecular Probes, Shanghai, China). HGC-27 cell apoptosis percentage was reflected by Annexin V/7-AAD percentage which was calculated by the number of Annexin V/7-AAD positive cells caught by fluorescence-activated cell sorting (FACS) (BECKMAN COULTER, Cytomics FC 500 MPL) divided by the total number of cells.

### Quantification of apoptosis by enzyme-linked immunosorbent assay (ELISA)

The Cell Apoptosis Histone-DNA ELISA Detection Kit (Roche, Palo Alto, CA) was used to quantify HGC-27 cell apoptosis with indicated treatment/s according to the manufacturer's protocol. Briefly, the cytoplasmic histone/DNA fragments from cells after treatments were extracted and bound to immobilized anti-histone antibody. Subsequently, the peroxidase-conjugated anti-DNA antibody was then added for the detection of immobilized histone/DNA fragments. After addition of substrate for peroxidase, the spectrophotometric absorbance of the samples was determined using a plate reader at a test wavelength of 405 nm.

### Beclin-1 RNAi Interference (RNAi) in HGC-27 cells

SiRNA for beclin-1 was purchased from Santa Cruz Biotech (sc-29797, Santa Cruz, CA). HGC-27 cells were cultured in complete medium (no antibiotic) and were seeded in a six-well plate one day before transfection and were 60% confluence on the following day. For RNAi experiments, 4.0 µl PLUS™ reagent (Invitrogen, Carlsbad, CA) was diluted at 90 µl of RNA dilution water (Santa Cruz, CA) for 5 min at room temperature. Then, 20 µl siRNA (10 µM) was added to PLUS™ reagent for 5 min in room temperature. Lipofectamine ™ (4.0 µl) (Invitrogen, Carlsbad, CA) was then added to the complex. After 30-min incubation at room temperature, the transfection complex was added to each well containing 1 ml of medium (MEM, no antibiotic, no FBS) with the final beclin-1 siRNA concentration of 200 nM. Cells were switched back to complete medium (10% FBS in opi-MEM, no anti-biotic) 12 h after transfection, and cultured for additional 48 h. Same concentration of scrambled control siRNA (Santa Cruz, 200 nM) was added to control cells. Beclin-1 protein expression and loading control were determined by western-blot to insure RNAi efficiency, only beclin-1 knockdown cells were used for further experiments.

### Immunochemistry

Cells were grown as monolayers on coverslips. Cells were fixed in −20°C cold methanol for 15 min, washed three times with PBS, and blocked with 1% BSA in PBS (pH 7.5) for 30 min followed by overnight incubation with primary antibody (anti-LC3B, cell signaling tech,1∶50) at 4°C. Secondary fluorescent FITC or Cy_3_ (Dako) was used at dilution 1∶500 in blocking buffer for 1 h. Cells were then incubated with DAPI (WeiAo, Shanghai) for 8 min and visualized by using a Nikon microscope fitted with the appropriate filters. The number of LC3B puncta positive cells in every 100 cells was recorded. Each condition was repeated 5 times.

### Statistical analysis

The data presented in this study were means ±standard deviation (SD). Statistical differences were analyzed by one-way *ANOVA* followed by multiple comparisons performed with post hoc Bonferroni test (SPSS version 14). Values of *p*<0.05 were considered statistically significant. The significance of any differences between two groups was tested using paired-samples *t* test when appropriated. CalcuSyn software (Version 2.0) was obtained from Researchsoft.com.cn (Beijing, China), and combination index (CI)<1 indicates synergism.

## Results

### RAD001 inhibits cell growth in multiple human gastric cancer cell lines

**Figure 1:**
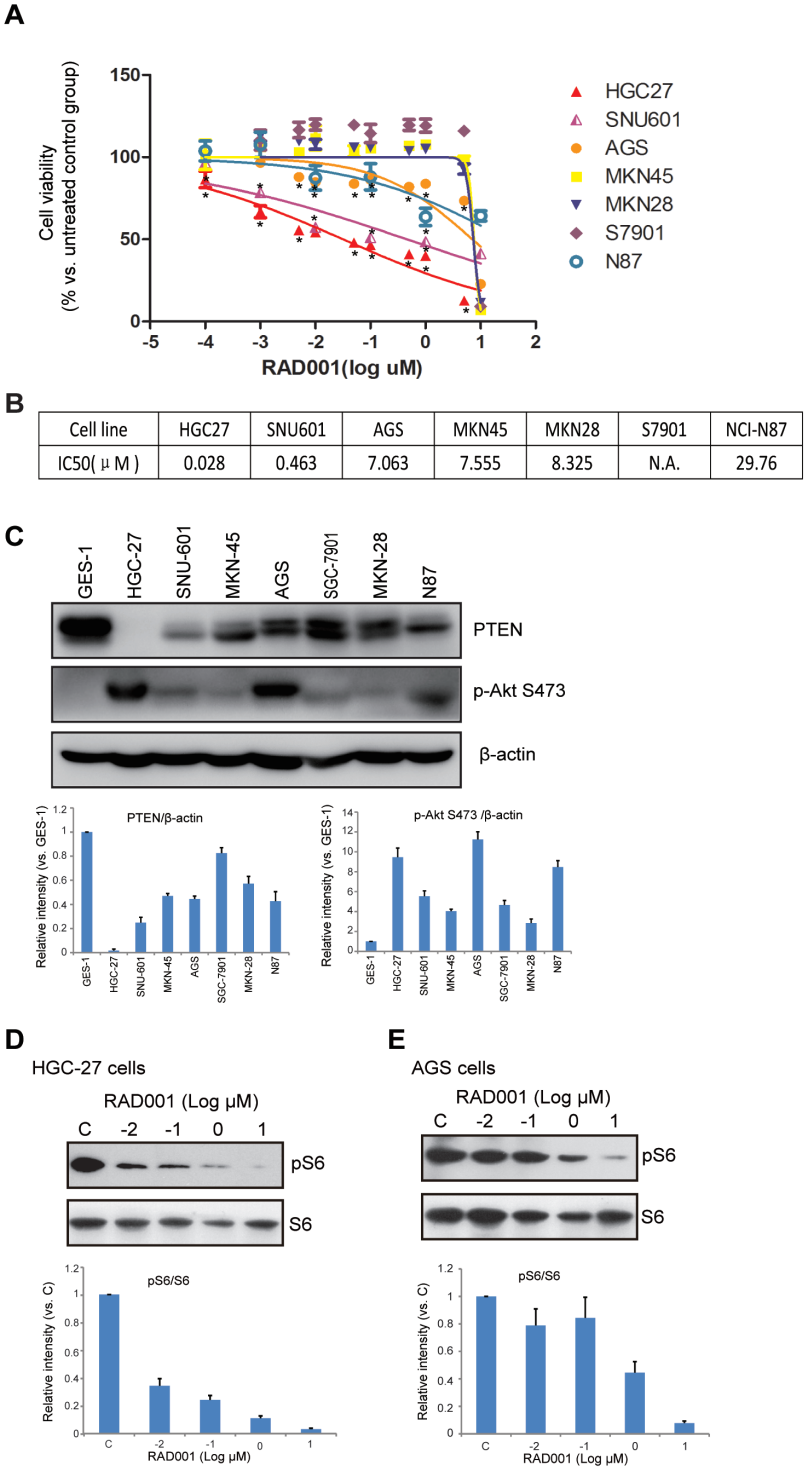
RAD001 inhibits cell growth in multiple human gastric cancer cell lines. Cultured gastric cancer cell lines AGS, MNK-45, HGC-27, SNU-601, MKN-28, SGC-7901 and N-87 were treated with different concentration of RAD001 for 72 h, afterwards, cell growth was detected by CCK-8 cell viability assay (A). IC-50 of RAD001 in these cell lines was shown (B). The expression level of PTEN, pAKT (Ser 473) and β-actin (equal loading) in above cell lines and GES-1 cells were also detected by western blot, PTEN and pAKT level was quantified as described (C). AGS and HGC-27 cells were treated with different concentration of RAD001 for 24 hours, phospho- and total S6 were detected by Western blot, pS6 level was quantified as described (D and E), and the number was normalized to the band labeled with “1.00”. The data shown was the mean from three independent experiments. *p<0.05.

We first examined the activity of RAD001 on cell growth in gastric cancer cell lines representing different genetic backgrounds, including AGS, MNK-45, HGC-27, SNU-601, MKN-28, SGC-7901 and N-87. Gastric cancer cell growth was reflected by cell viability which was detected by CCK-8 assay. RAD001 inhibited cell growth in all gastric cancer cells, as the cell viability OD decreased after different concentrations of RAD001 treatment (Figure 1A, Figure S1A and B). However, these different lines showed different sensitivity to RAD001. HGC-27 and SNU-601,were the most sensitive ones (Figure 1A, Figure S1A and B). IC-50s of RAD001 in these different lines were also presented. HGC-27 and SNU-601 had the lowest IC-50, which further confirmed their highest sensitivity to RAD001 (Figure 1B,). Western blot results in Figure 1C showed the expression of PTEN and p-AKT (Ser 473) in above gastric cancer cells. Results in Figure 1C show that SNU-601 cells expressed extremely low level of PTEN, which was also supported by paper by Byun DS et al [22]. Results indicated that RAD001-sensitive lines were cells with no or low expression level of PTEN (HGC and SNU601). More ever, HGC-27 and AGS were both sensitive to RAD001 on mTOR (pS6) inhibition (Figure 1D and E).

### RAD001 and MK-2206 synergistically inhibits the growth of HGC-27 and SNU-601 cells

**Figure 2:**
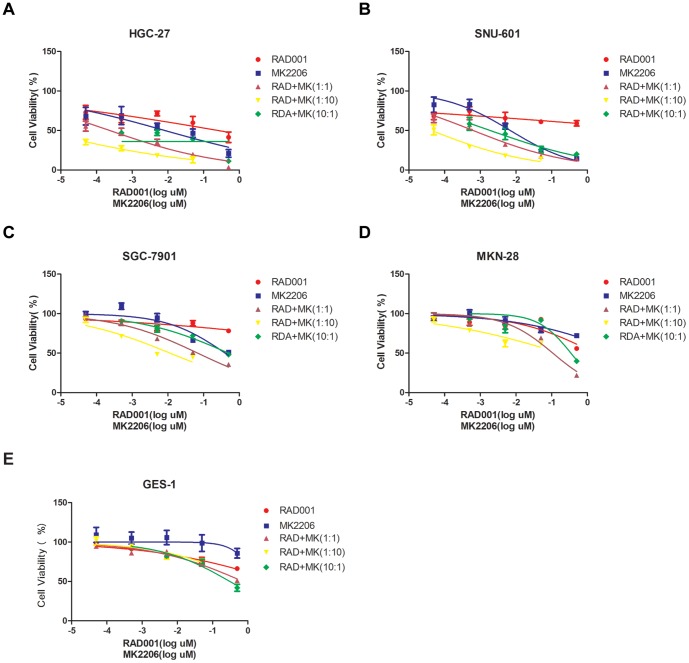
RAD001 and MK-2206 synergistically inhibits the growth of HGC-27 and SNU-601 cells. HGC-27, SNU-601, SGC-7901, MKN-28 and GES-1 cells were treated for 72 h with different concentration of RAD001 and/or MK-2206 with different ratio. Inhibition of cell growth was measured by CCK-8 cell viability assay (A–E). The data shown are the mean from three independent experiments, each with six wells.

The main object of this current study is to test the synergistic anti-gastric cancer cell ability of RAD001 and MK-2206. CCK-8 cell viability results in Figure 2A and B demonstrated that either RAD001 or MK-2206 alone had a moderate effect on HGC-27 and SNU-601 cell growth, however, combination of the two at a relative lower concentration significantly inhibited the growth of both cells, as the CCK-8 OD value decreased dramatically in cells treated with both agents (Figure 2A and B). Further, RAD001 and MK-2206 at ratio 1∶10 showed most significant synergistic effects (Figure 2A and B). The computer software Calcusyn was used to test combination index (CI) between RAD001 and MK-2206 [23], CI<1.0 was considered as synergism [23], as seen in Figure S1C and D. Hence, the combination of RAD001 and MK-2206 showed synergistic inhibitory activity on HGC-27 and SNU-601 cell growth *in vitro*. Results in Figure S1E showed that RAD001 and MK-2206 synergistically induced HGC-27 cell death, as the number of trypan blue cells (“dead” cells) increased significantly after the co-administration, similar results were also obtained in SNU-601 cells (data not shown). We repeated the same treatment (RAD001 plus MK-2206 combination) in SGC-7901, GES-1 cells (high PTEN expression) and MKN-28 cells(middle PTEN expression). Results clearly showed that the synergistic effects was most significant in low-PTEN expression cells (HGC-27 cells and SNU-601 cells) (Figure 2A–B), but was least significant in SGC-7901, GES-1 cells (high PTEN expression) (Figure 2C, 2E). Figure S1E showed the combination had no significant synergistic effect on GES-1.The effect in MKN-28 cells was mediocre (Figure 2D). These results indicate that PTEN level is a determinate factor of the synergism efficiency, and the synergistic effect is most significant in low-PTEN cells.

### RAD001 and MK-2206 together induces HGC-27 cell G1/S arrest

**Figure 3:**
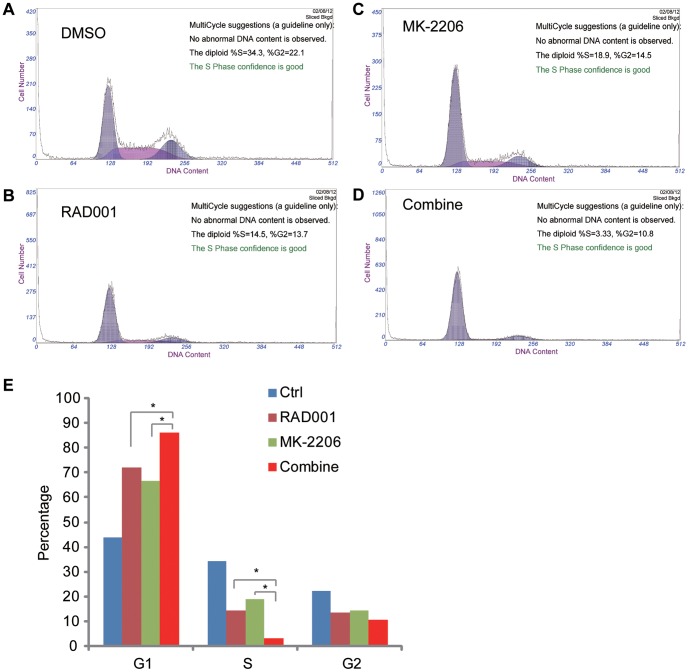
RAD001 and MK-2206 together induces HGC-27 cell G1/S arrest. HGC-27 cells were treated for 48 h with RAD001 (10 nM) and/or MK-2206 (100 nM), cell cycle was analyzed by FACS as described (A–D). (E) summarized the HGC-27 cell cycle distributions after RAD001 (10 nM) and/or MK-2206 (100 nM) treatment. The results in this figure were from repeated three independent experiments, with similar results obtained. *p<0.05.

The effects of RAD001 and/or MK-2206 on HGC-27 cell cycle progression were examined. Results in Figure 3A–D showed that either RAD001 or MK-2206 alone exerted moderate effect on HGC-27 cell cycle progression, while the combination of the two agents induced a profound G1/S arrest. The statistic data in Figure 3E demonstrated that the RAD001 plus MK-2206 treatment group had the highest percentage of G1 phase cells and the lowest percentage of S phase cells. These data suggested that the combination of RAD001 and MK-2206 together induces HGC-27 cell G1/S arrest.

### The combination of RAD001 and MK-2206 fails to induce significant apoptosis in HGC-27 cells

**Figure 4:**
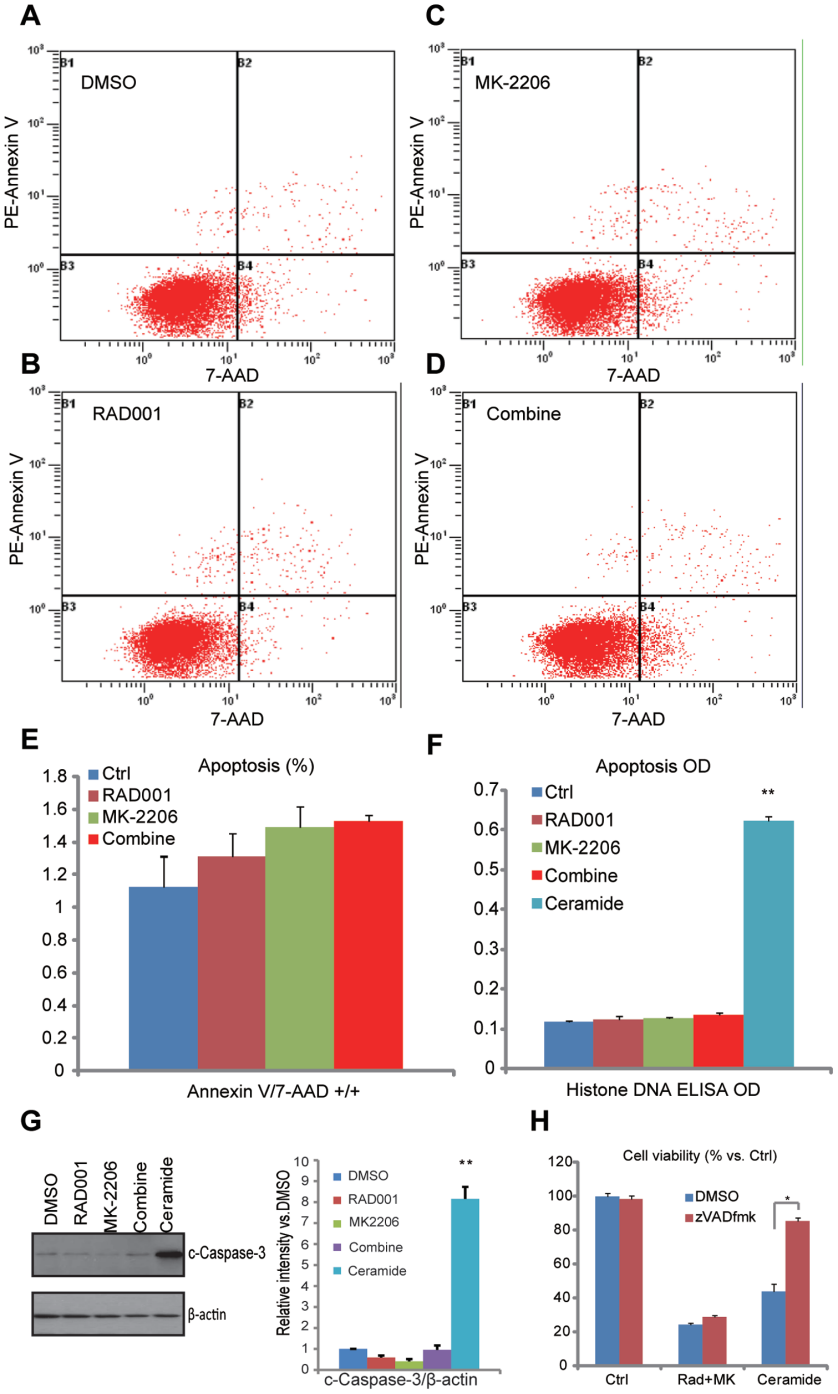
The combination of RAD001 and MK-2206 fails to induce significant apoptosis in HGC-27cells. HGC-27 cells were treated for 24 h with RAD001 (10 nM) and/or MK-2206 (100 nM), cell apoptosis was detected by FACS sorting positive Annexin V/7-AAD positive cells (A–E), apoptosis ELISA assay (F) and western blots detecting cleaved caspase-3 (G). C6 ceramide (10 µg/ml, 24 h)-induced cleaved caspase-3 was tested by western blot (G). HGC-27 cells were pretreated with general caspase-3 inhibitor zVADfmk (40 µM) for 1 hours, followed by RAD001 (10 nM) plus MK-2206 (100 nM) administration, cell viability was examined by CCK-8 assay 72 h after treatment/s (H). The data shown are the mean from three independent experiments. ** *p*<0.05 vs. Ctrl group or DMSO group. (F, G). *p<0.05 (H).

The above results showed that co-administration of RAD001 and MK-2206 induced synergistic growth inhibition, cell cycle arrest and HGC-27 cell death, so we then tested whether cell apoptosis was involved in the process. HGC-27 cell apoptosis was tested by three different assays including FACS sorting Annexin V/7-AAD positive cells (Figure 4A–E), histone-DNA apoptosis ELISA assay (Figure 4F) [24] and western blots detecting cleaved-caspase-3 (Figure 4G). Intriguingly, results from these assays showed that there was no significant cell apoptosis induced by RAD001 and/or MK-2206 in HGC-27 cells (Figure 4A–G), suggesting that apoptosis may not be required for the cytotoxic effects by the combination. As a matter of fact, general caspase inhibitor z-VAD-fmk failed to rescue HGC-27 cell viability loss by RAD001 and MK-2206 (Figure 4H). On the other hand, C6-ceramide, an apoptosis intermediate player [24], induced significant cell apoptosis (Figure 4F and G) and cell viability loss (Figure 4H) in HGC-27 cells, which was almost reversed by z-VAD-fmk (Figure 4H).

### Autophagic cell death is important for the cytotoxic effects induced by RAD001 and MK-2206 co-administration

**Figure 5:**
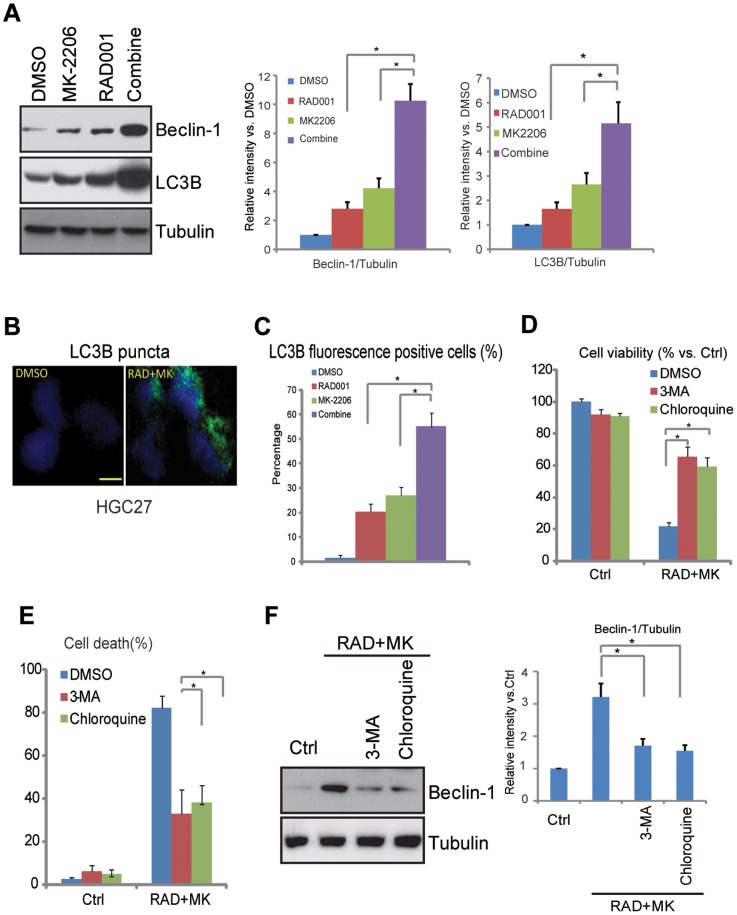
Autophagic cell death is important for the cytotoxic effects by RAD001 and MK-2206 co-administration. HGC-27 cells were treated for 48 h with RAD001 (10 nM) and/or MK-2206 (100 nM), expression levels of LC3B and beclin-1 were determined by western blots (A). LC3B puncta was tested by immuno-fluorescence as described (B and quantified in C). HGC-27 cells were pre-treated with autophagy inhibitors 3-MA (10 mM) or hydroxychloroquine (20 mM) for 1 hour, followed by RAD001 (10 nM) and MK-2206 (100 nM) treatment, afterwards, expression level beclin-1 and tubulin was determined by western blots after 48 hours (F), cell viability was analyzed by CCK-8 assay 72 hours after treatment (D), trypan positive cells were also recorded (E). The data shown are the mean from three independent experiments. *p<0.05.

The above results showed that apoptosis is not induced by RAD001 and/or MK-2206, thus, the potential role of autophagy on the cytotoxic effects by RAD001 and MK-2206 was tested. We observed a significant autophagy induction after RAD001 and MK-2206 co-treatment in HGC-27 cells, as light chain 3B (LC3B) and beclin-1, two key indicators of cell autophagy [25], [26], [27], increased significantly after the co-administration (Figure 5A and B). Either drug alone also enhanced their expressions at a lower degree (Figure 5A). LC3B puncta was also examined by immuno-fluorescence (Figure 5B) and the number of LC3B puncta cells after corresponding treatment was recorded (Figure 5C). Two autophagy inhibitors 3-methyladenine (3-MA) [28] and chloroquine [29], which inhibited RAD001 plus MK-2206-induced beclin-1 expression (Figure 5F), significantly inhibited cell viability loss (Figure 5D) and cell death (Figure 5E) by the co-administration, suggesting that autophagic cell death is required for the cytotoxic effects seen in the synergic combination. Note that 3-MA and chloroquine also inhibited cell viability loss by RAD001 or MK-2206 alone in HGC-27 cells (Figure S2).LC3B puncta was also examined on SNU-601 cells, and the result was unanimous with which on HGC-27 cells (Figure S3).

### The combination of RAD001 and MK-2206 exerts enhanced effects on Akt/mTOR suppression and cyclin D1 down-regulation

**Figure 6:**
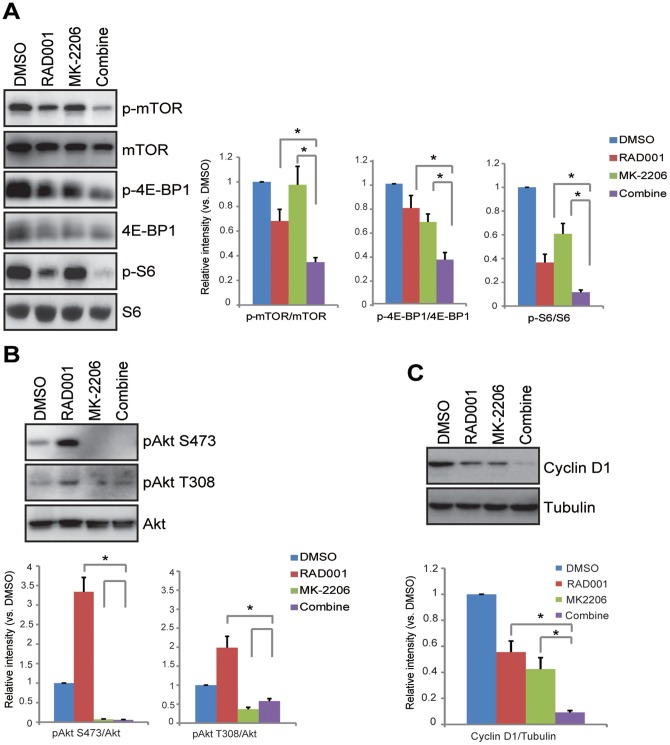
The combination of RAD001 and MK-2206 exerts enhanced effects on Akt/mTOR suppression and cyclin D1 down-regulation. HGC-27 cells were treated for 24 h with RAD001 (10 nM) and/or MK-2206 (100 nM), phospho- and total levels of Akt, mTOR, S6 and 4E-BP1 (A and B) as well as expression levels of cyclin D1 and β-actin (C) were detected by western blots. The relative expression level of p-mTOR, p-S6, p-4E-BP1, p-Akt (Ser 473, Thr 308) and cyclin D1 was quantified as described; the number was expressed as fold change vs. the corresponding band of DMSO. Experiments in this figure were repeated three times and similar results were obtained. *p<0.05.

We then examined signaling changes in HGC-27 cells after RAD001 and/or MK-2206 treatment/s using western blots by focusing on Akt/mTOR signaling. Phosphorylation of mTOR (Ser 2448), S6 (Ser 235/236) and 4E-BP1 (Ser 65) were used as indicators of mTOR complex 1 (mTORC1) activation [9], [10], [30]. As shown in Figure 6A, either RAD001 or MK-2206 slightly inhibited mTORC1 activation in HGC-27 cells. Significantly, the combination of the two exerted enhanced effects on mTORC1 suppression (see quantified results). Notably RAD001 induced Akt activation while inhibitor mTOR in HGC-27 cells, as phosphorylation Akt (Ser 473 and Thr 308) were increased after RAD001 treatment (Figure 6B). Meanwhile, MK-2206, the Akt inhibitor, blocked basal and RAD001-induced Akt activation (Figure 6B) (see quantified results). The combination of RAD001 and MK-2206 also exerted enhanced effects on cyclin D1 down-regulation (Figure 6C) (see quantified results). These results suggested that the combination of RAD001 and MK-2206 synergistically inhibited Akt/mTOR activation and cyclin D1 expression, which might be responsible for growth inhibition.

### ERK/MAPK-dependent beclin-1 expression is important for autophagic cell death by RAD001 and MK-2206

**Figure 7:**
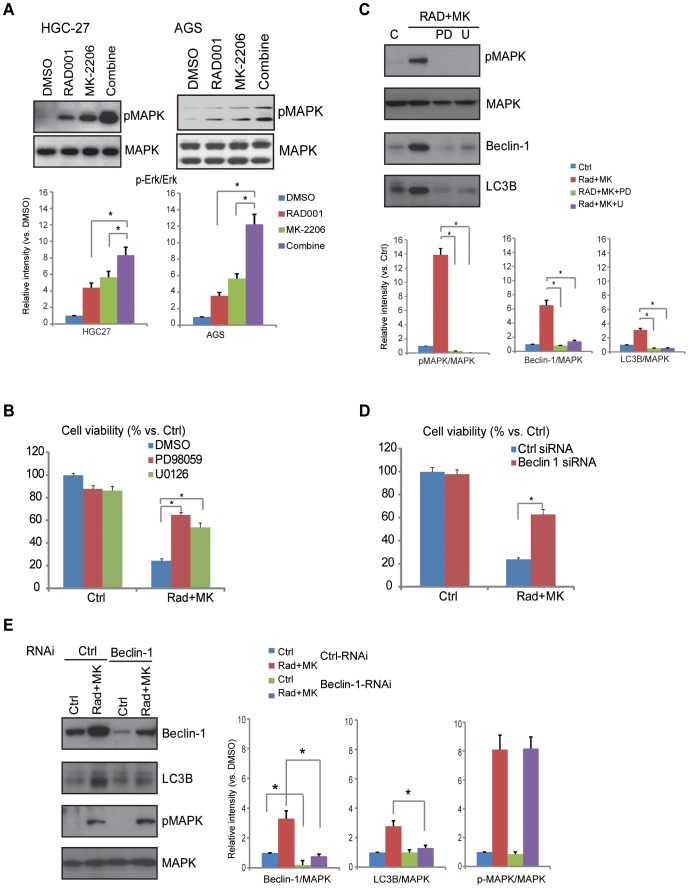
ERK/MAPK-dependent beclin-1 expression is important for autophagic cell death induced by RAD001 and MK-2206. HGC-27 cells and AGS cells were treated for 24 h with RAD001 (10 nM) and/or MK-2206 (100 nM), phospho- and total levels of ERK/MAPK were tested by western blots (A). HGC-27 cells were pre-treated with MEK/ERK inhibitors PD98059 (PD, 1 µM) or U0126 (U, 1 µM) for 1 hour, followed by RAD001 (10 nM) and MK-2206 (100 nM) co-administration, 72 h later, cell viability was analyzed (B), the expression levels of beclin-1 and LC3B were also examined by western blots 48 h after administration. Scramble and beclin-1 siRNA transfected HGC-27 cells were treated with RAD001 (10 nM) and MK-2206 (100 nM) co-administration, cell viability (72 h after treatment) was measured by CCK-8 assay (D), and expression of LC3B, beclin-1, phospho- and total-MAPK were tested by western blots (E, after 48 h). p-MAPK (A and E), Beclin-1 (E) and LC3B (E) were quantified the same as above, and the number was normalized to the corresponding band of control. The data shown are the mean from three independent experiments. *p<0.05.

In an effect to test the potential role of RAD001 and/or MK-2206 on MAPK activation in HGC-27 and AGS cells (low PTEN expression cells), we observed a significant ERK/MAPK activation by RAD001 or MK-2206. Further, the combination of the two agents exerted enhanced effects on ERK/MAPK activation (Figure 7A) (see quantified results). No significant activations of p38 or JNK, two other cascades in MAPK signaling, were observed by RAD001 and/or MK-2206 (data not shown). The fact that PD98059 and U0126, two MEK/ERK inhibitors, suppressed RAD001 plus MK-2206-induced cell viability loss suggested that ERK/MAPK activation is important for the cytotoxicity by the co-administration (Figure 7B). Significantly, PD98059 and U0126 also inhibited beclin-1 and LC3B induction by RAD001 and MK-2206 co-administration to suggest that ERK/MAPK activation is required for autophagy induction in this situation (Figure 7C). Furthermore, RNAi knockdown of beclin-1 inhibited LC3B induction (Figure 7E) and viability loss (Figure 7D) by RAD001 plus MK-2206, without affecting MAPK activation (Figure 7E). Based on these data, we suggested that ERK/MAPK-dependent beclin-1 expression is important for autophagic cell death by RAD001 and MK-2206 synergism.

## Discussion

Inhibition of oncogenic signaling i.e. Akt/mTOR with targeted small molecule inhibitors is a powerful therapeutic approach to treat gastric cancer and possibly other solid tumors [9], [10]. These inhibitors are efficacious as a single agent in some cases, but improved anti-cancer activities can often be achieved by combining them with other anti-cancer agents [31]. For example, enhanced anti-tumor activity was reported by combining specific Akt and mTOR inhibitors [32], [33]. This current study shows that MK-2206 (an Akt inhibitor) and RAD001 (a mTOR inhibitor) can be combined to synergistically inhibit PI3K/Akt/mTOR signaling and gastric cancer cell growth. Our observations further support that lower dose of mTOR inhibitor (i.e. RAD001) can achieve full target inhibition when combined with an allosteric Akt inhibitor (i.e. MK-2206). Our results here are consistent with recent publications in other cancer cell lines [34], although here we proposed a novel synergistic autophagic cell death pathway by these two inhibitors.

As a single agent, RAD001 can only partially inhibits 4EBP1 T37/46 phosphorylation (also seen in our data) and cell proliferation [30], suggesting that it only partially affects mTOR signaling [35], [36]. Here, we also observed a partial inhibition of mTOR signaling by low-dose of RAD001 alone, however, the combination with MK-2206 caused an enhanced inhibitory effect on mTOR activation. Another important fact is that RAD001 is known to activate insulin receptor substrate 1 (IRS-1)-Akt signaling [16], [17], which is suggested to potentially attenuate its effects on tumor cell proliferation and viability [17]. In consistency with these studies, we observed a significant Akt activation (Ser 473 and Thr 308 phosphorylation) after RAD001 treatment in HGC-27 cells, and co-treatment with MK-2206 blocked RAD001-induced Akt activation. Together these data suggest that MK-2206 not only enhanced mTOR inhibition, but also blocked Akt activation by RAD001, which might explain the synergistic effects.

Interestingly, we failed to observe significant cell apoptosis by RAD001 and/or MK-2206 in HGC-27 cells, suggesting that apoptosis may not play a significant role in the cytotoxic effects of the combination. Rather, data from this study suggest that autophagic cell death is required for the cytotoxic effects of the combination. These data are not surprising, as mTOR inhibition is the major trigger of cell autophagy [37], [38], [39]. mTOR inhibition frees Ulk1 leading to its activation which triggers cell autophagy [37], [38], [39]. As a matter of fact, recent studies have suggested that autophagic cell death mediates anti-tumor ability by a number of mTOR inhibitors or AMP-activated protein kinase (AMPK) activators [37], [39], [40]. Both RAD001 and MK-2206 are able to induce cell autophagy as shown in many other cancer cells [18], [41], [42], [43]. Our study here suggests that RAD001 and MK-2206 synergistically inhibit mTOR activation, which might lead to autophagic, but not apoptotic cell death.

A recent study by Wang et al., suggests that although moderate or transit MEK/ERK activation might inhibit cell autophagy, sustained and profound MEK/ERK activation can actually induce autophagy [44]. In the same study, the authors found that MEK/ERK, as the downstream of AMPK, induces beclin-1-dependent autophagic cell death [44]. Even more intriguing, the authors suggest that mTOR inhibition (as seen here) is pre-required for the beclin-1/autophagy induction by MEK/ERK [44]. In the current study, we observed a profound ERK/MAPK activation by the co-administration, which appears important for RAD001 plus MK-2206-induced beclin-1 expression, autophagy induction and cytotoxic activity. However, this process is AMPK-independent, as no significant AMPK activation was observed in cells treated with RAD001 and/or MK-2206 (data not shown). Our results showing that Erk activation by Akt/mTOR inhibition were consistent with recent publications [45].

It should be noted that inhibition of the pathways mentioned above i.e. autophagy inhibition by 3-MA or chloroquine, ERK/MAPK inhibition by PD98059 or U0126 or beclin-1 inhibition by RNAi, didn't totally abolished RAD001 and MK-2206 co-administration-induced cytotoxic effects, this might due to incomplete inhibition by these inhibitors or RNAi. However, it is more likely that other undefined signaling mechanisms or cell death pathways may also be involved in the process, which obviously need further investigation. Future studies may also need to focus on the detailed cellular mechanism of ERK/MAPK activation and autophagy induction by the co-administration.

## Supporting Information

Figure S1 **Response of HGC-27 and SNU-601 cells to RAD001 and/or MK-2206.** Cultured gastric cancer cell lines HGC-27 and SNU-601 were treated with different concentration of RAD001 (starting at 10-7 µM) for 72 h, afterwards, cell growth was detected by CCK-8 cell viability assay (A and B). The “Calcusyn” software was applied to calculate combination index (CI) between each RAD001 and/or MK-2206 dose combination for the CCK-8 data obtained from HGC-27,SNU-601 and GES-1 cells (see Figure 2A, B and E). CI<1 was considered as synergism(C-D). HGC-27 cells were treated for 72 h with RAD001 (10 nM) and/or MK-2206 (100 nM), trypan blue staining was applied to stain “dead” cells (E). The data shown are the mean from three independent experiments. *p<0.05.
(TIF)

Figure S2 **3-MA and chloroquine inhibits cell viability loss by RAD001 or MK-2206 as a single agent in HGC-27 cells.** (A) HGC-27 cells were pre-treated with autophagy inhibitors 3-MA (10 mM) or hydroxychloroquine (20 mM) for 1 hour, followed by 72 hours of RAD001 (10 nM) or MK-2206 (100 nM) treatment, afterwards, cell viability was analyzed by CCK-8 assay. The data shown are the mean from three independent experiments, each with six wells. *p<0.05.
(TIF)

Figure S3 **LC3B expression induced by RAD and/or MK2206 on SNU-601 cells.** SNU-601 cells were treated with RAD001 (10 nM) and/or MK-2206 (100 nM) for 48 h. LC3B puncta was tested by immune-fluoresence as described (A and quantified in B). The data shown are the mean from three independent experiments. *p<0.05.
(TIF)
