# Supplementary figures and images for: The Combination of RAD001 and MK-2206 Exerts Synergistic Cytotoxic Effects against PTEN Mutant Gastric Cancer Cells: Involvement of MAPK-Dependent Autophagic, but Not Apoptotic Cell Death Pathway
Source: PLoS One. 2014 Jan 9;9(1):e85116. doi: 10.1371/journal.pone.0085116 (PMC3887024; doi:10.1371/journal.pone.0085116)

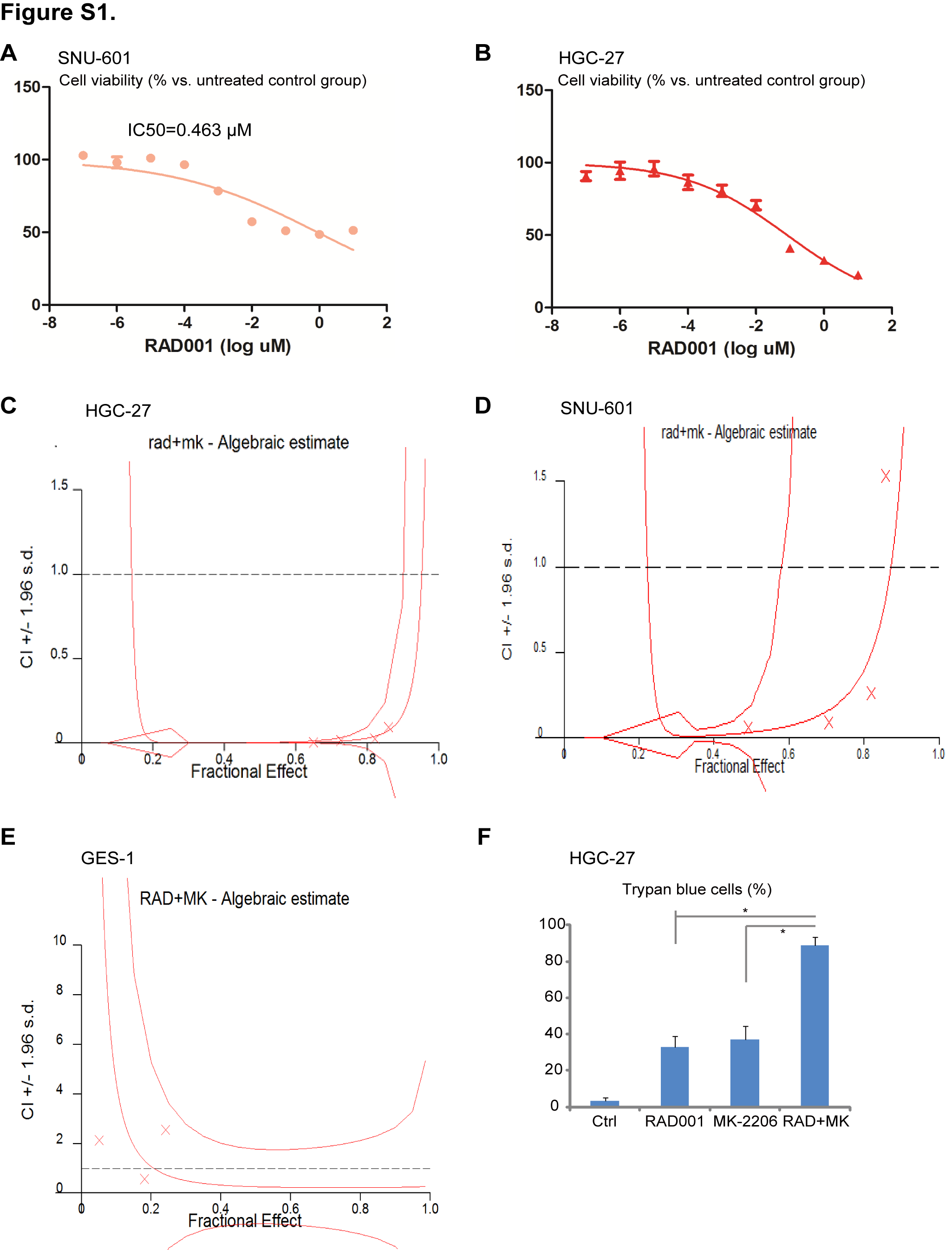

Supplement: Figure S1 — Response of HGC-27 and SNU-601 cells to RAD001 and/or MK-2206. Cultured gastric cancer cell lines HGC-27 and SNU-601 were treated with different concentration of RAD001 (starting at 10-7 µM) for 72 h, afterwards, cell growth was detected by CCK-8 cell viability assay (A and B). The “Calcusyn” software was applied to calculate combination index (CI) between each RAD001 and/or MK-2206 dose combination for the CCK-8 data obtained from HGC-27,SNU-601 and GES-1 cells (see Figure 2A, B and E). CI<1 was considered as synergism(C-D). HGC-27 cells were treated for 72 h with RAD001 (10 nM) and/or MK-2206 (100 nM), trypan blue staining was applied to stain “dead” cells (E). The data shown are the mean from three independent experiments. *p<0.05. (TIF) [file pone.0085116.s001.tif]

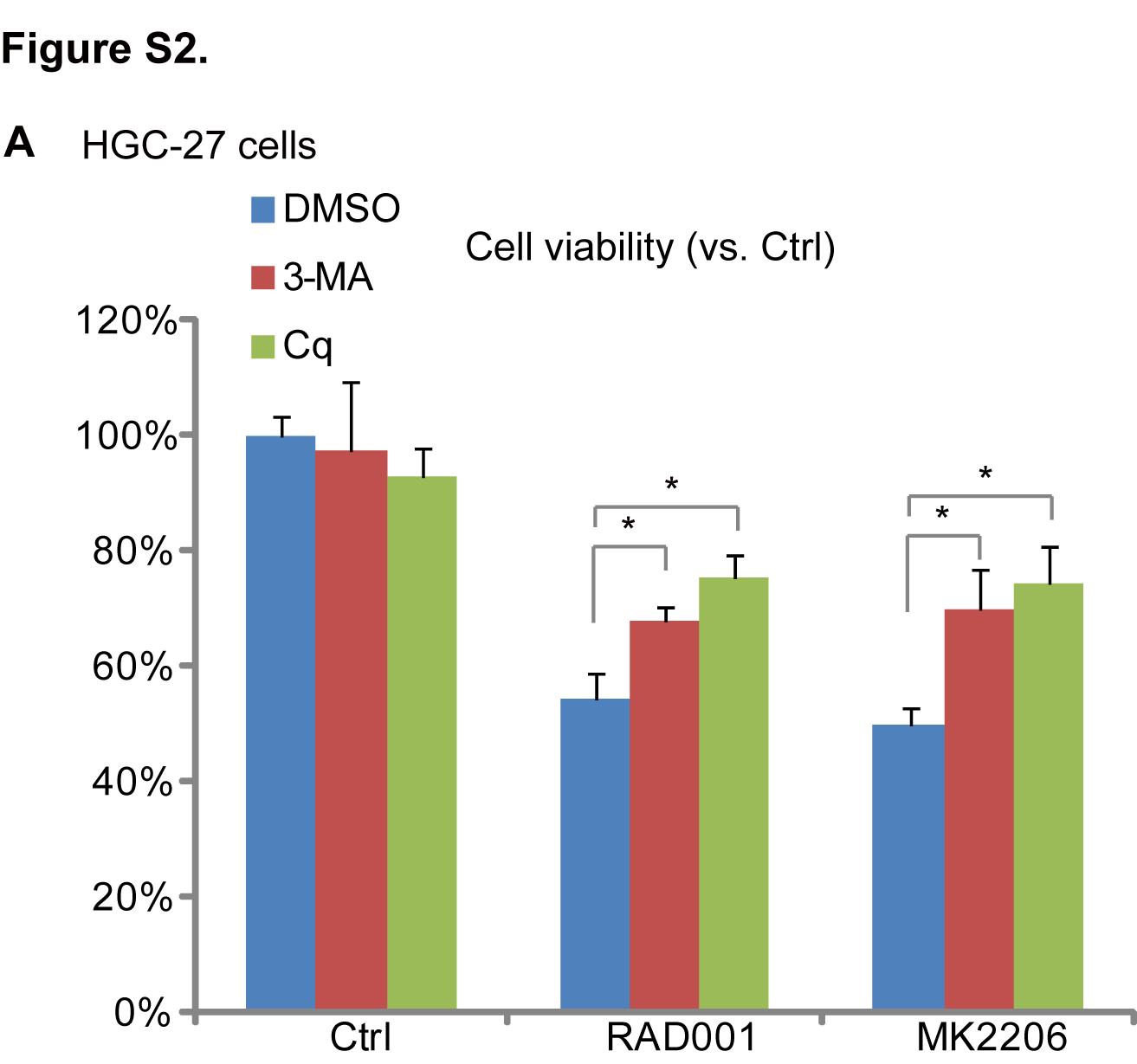

Supplement: Figure S2 — 3-MA and chloroquine inhibits cell viability loss by RAD001 or MK-2206 as a single agent in HGC-27 cells. (A) HGC-27 cells were pre-treated with autophagy inhibitors 3-MA (10 mM) or hydroxychloroquine (20 mM) for 1 hour, followed by 72 hours of RAD001 (10 nM) or MK-2206 (100 nM) treatment, afterwards, cell viability was analyzed by CCK-8 assay. The data shown are the mean from three independent experiments, each with six wells. *p<0.05. (TIF) [file pone.0085116.s002.tif]

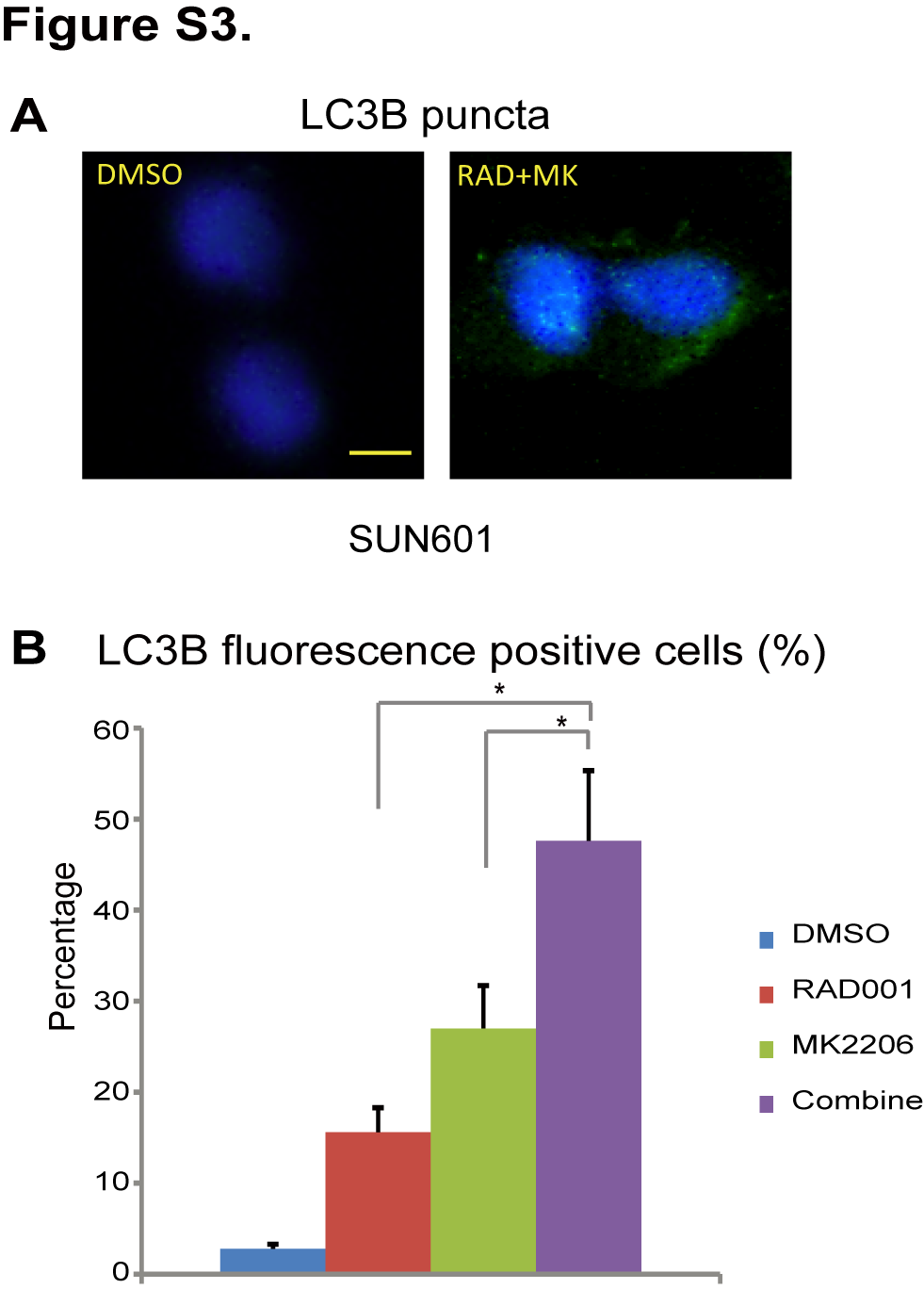

Supplement: Figure S3 — LC3B expression induced by RAD and/or MK2206 on SNU-601 cells. SNU-601 cells were treated with RAD001 (10 nM) and/or MK-2206 (100 nM) for 48 h. LC3B puncta was tested by immune-fluoresence as described (A and quantified in B). The data shown are the mean from three independent experiments. *p<0.05. (TIF) [file pone.0085116.s003.tif]
